# Microcystic, elongated and fragmented (MELF) pattern in endometrial carcinoma: clinicopathologic analysis and prognostic implications

**DOI:** 10.1097/MD.0000000000031369

**Published:** 2022-10-28

**Authors:** Jinghua Song, Huajun Li, Hongyan Guo, Yuhan Cai

**Affiliations:** a Center for Reproductive Medicine, Department of Obstetrics and Gynecology, Peking University Third Hospital, Beijing, China; b National Clinical Research Center for Obstetrics and Gynecology, Peking University Third Hospital, Beijing, China; c Key Laboratory of Assisted Reproduction, Peking University, Ministry of Education, Beijing, China; d Beijing Key Laboratory of Reproductive Endocrinology and Assisted Reproductive Technology, Peking University Third Hospital, Beijing, China.

**Keywords:** lymph node metastasis, elongated, endometrial endometrioid carcinoma, “microcystic, and fragmented (MELF)” pattern, mismatch repair

## Abstract

To assess the clinical value of microcystic, elongated, and fragmented (MELF) pattern in Chinese patients with endometrial endometrioid carcinoma. A total of 189 patients with endometrial endometrioid carcinoma were retrospectively analyzed in Peking University Third Hospital from January 2017 to December 2019. We analyzed the association of MELF pattern with the histopathologic data and prognosis of the patients, while immunohistochemistry was performed. The frequency of MELF pattern was 17.99% (34/189). MELF pattern was associated significantly with tumor size, myometrial invasion, histological grade, International Federation of Gynecology and Obstetrics stages, lymphovascular space invasion, and lymph node metastasis. According to multivariate logistic regression analysis, lymphovascular space invasion [95% confidence interval 1.021–48.485, *P* = .048] was a significant predictor of lymph node involvement. However, MELF pattern was not a significant predictor (95% confidence interval 0.054–2.279, *P* = .400). Loss of expression for mismatch repair proteins was observed in 10 MELF + cases (29.41%) and 54 MELF− cases (34.84%), respectively. All patients were followed up for 36.8 ± 8.9 months (18–54 months). Only 1 patient with MELF pattern was diagnosed with vaginal recurrence 28 months after the surgery. MELF pattern was associated with adverse histologic findings in endometrial endometrioid carcinomas. However, MELF pattern was statistically not a valuable predictor of lymph node metastasis and it needs more studies to show whether MELF pattern has an impact on the prognosis of patients with endometrial endometrioid carcinoma. MELF pattern may be important for identifying those patients who need comprehensive staging surgery.

## 1. Introduction

Endometrial carcinoma is the most frequent gynecological neoplasia in women, with an increase in incidence and mortality over the past few decades. Endometrial endometrioid carcinoma is the most common histologic subtype. Although patients with low-grade (grades 1 and 2) endometrioid carcinoma have better outcomes than those with high-grade (grade 3) endometrioid carcinoma,^[1]^ a subset of patients with low-grade endometrioid carcinoma have a recurrence and adverse prognosis. Therefore, there is a constant need for novel prognostic factors which may improve patient risk stratification. Among these, microcystic, elongated, and fragmented (MELF) pattern of myometrial invasion has recently been related to increased risk of lymphovascular space invasion (LVSI), lymph node metastasis (LNM) and extra-uterine disease.^[2–5]^ However, the biological and prognostic significance of MELF pattern in endometrial endometrioid carcinomas remains uncertain.

This study was conducted to elucidate clinicopathologic features and the prognostic value of MELF pattern in Chinese patients with endometrial endometrioid carcinoma. For that purpose, we retrospectively reviewed data of 189 consecutive patients with endometrial endometrioid carcinoma and analyzed the clinicopathologic and prognostic features. Furthermore, some immunohistochemical analyses were applied to elucidate the nature of tumor cells in MELF pattern.

## 2. Materials and methods

### 2.1. Study population and clinical information

This study examined 189 consecutive patients with endometrial endometrioid carcinoma that were resected with total hysterectomy and bilateral salpingo-oophorectomy with or without lymphadenectomy at the Department of Obstetrics and Gynecology, Peking University Third Hospital (Beijing, China), between January 2017 and December 2019. All patients provided written informed consent according to institutional guidelines. Patients were informed that the resected specimens were going to be stored by the Pathology Unit of the Peking University Third Hospital and might potentially be used for scientific research, and that their privacy would be maintained. Clinical and demographic information was collected from patient charts, including age at surgery, adjuvant chemotherapy and/or radiation therapy, recurrence and survival status.

### 2.2. Histopathologic evaluation and immunohistochemistry (IHC)

In all cases, 2 experienced gynaecological pathologists established the histological diagnosis of endometrial endometrioid carcinoma after an extensive and careful evaluation of tumor specimens, according to the WHO Classification of Tumours of Female Reproductive Organs.^[6]^ Histopathologic findings, including the extent of myometrial invasion, invasion to the uterine cervix, LVSI and LNM, were evaluated to confirm the initial diagnosis. Finally, the International Federation of Gynecology and Obstetrics (FIGO) staging system published in 2009 was applied to all patients. Patients with mixed carcinoma (endometrioid carcinoma and serous/clear cell carcinoma) were excluded because high-grade components can influence patient survival.^[7]^

IHC was performed with the labeled streptavidin–biotin peroxidase detection system. Mismatch repair (MMR) proteins status was determined with the antibodies (MLH1, MSH2, MSH6 and PMS2) in the setting of intact control stromal/lymphocyte staining. Cases were considered as showing stable immunophenotype (MMR+) if any tumor cell nuclei showed positive staining, and unstable immunophenotype (MMR−) if all tumor cell nuclei were negative in the presence of internal positive control immunoreactivity. Stromal/lymphocyte staining as well as nonneoplastic endometrial glands were used as positive internal controls. The expression profile of p53 was evaluated by estimating the proportion of nuclear staining of tumor cells. Cases in which nuclear staining was observed in at least 10% of cancer cells were classified as a p53-stained group. Cases were classified as “p53 wild type” (p53-wt: focal and/or heterogeneous staining pattern) and “p53 immunohistochemically mutated” (diffuse expression in at least 75% of tumor cell nuclei); cases showing complete absence of staining in tumoral nuclei were considered as “null phenotype”.

### 2.3. Assessment of MELF pattern

MELF pattern was initially recognized by Lee, Vacek and Belinson,^[8]^ but the term MELF was first defined by Murray et al^[9]^ The histological appearance of invasive glands, as cystic-dilated or slit-like, lined by flattened, endothelial-like epithelium or squamoid tumor cells, with eosinophilic cytoplasm, often with intraluminal tufts or fragmented, alongside with small groups or isolated tumor cells, led to their denomination as “microcystic, elongated and fragmented glands”.

### 2.4. Statistical analysis

All statistical analyses were performed using SPSS 22.0 for Windows (IBM SPSS Statistics, IBM software, Armonk, NY). Values were given as mean ± standard deviation (SD) or median (interquartile range). Continuous variables were tested for normality by the Kolmogorov–Smirnov test. The analysis of the differences between groups was assessed by the Welch *t* test or the Mann-Whitney-Wilcoxon test for parametric or nonparametric data respectively. The Chi-square test was applied to compare proportions of categorical variables. The multivariate logistic regression analysis was used to study the possible correlation between MELF pattern and risk of lymph node metastasis. Significance was defined as *P* < .05.

## 3. Results

### 3.1. Clinical characteristics and clinicopathologic parameters of endometrial endometrioid carcinoma

**Figure 1.**
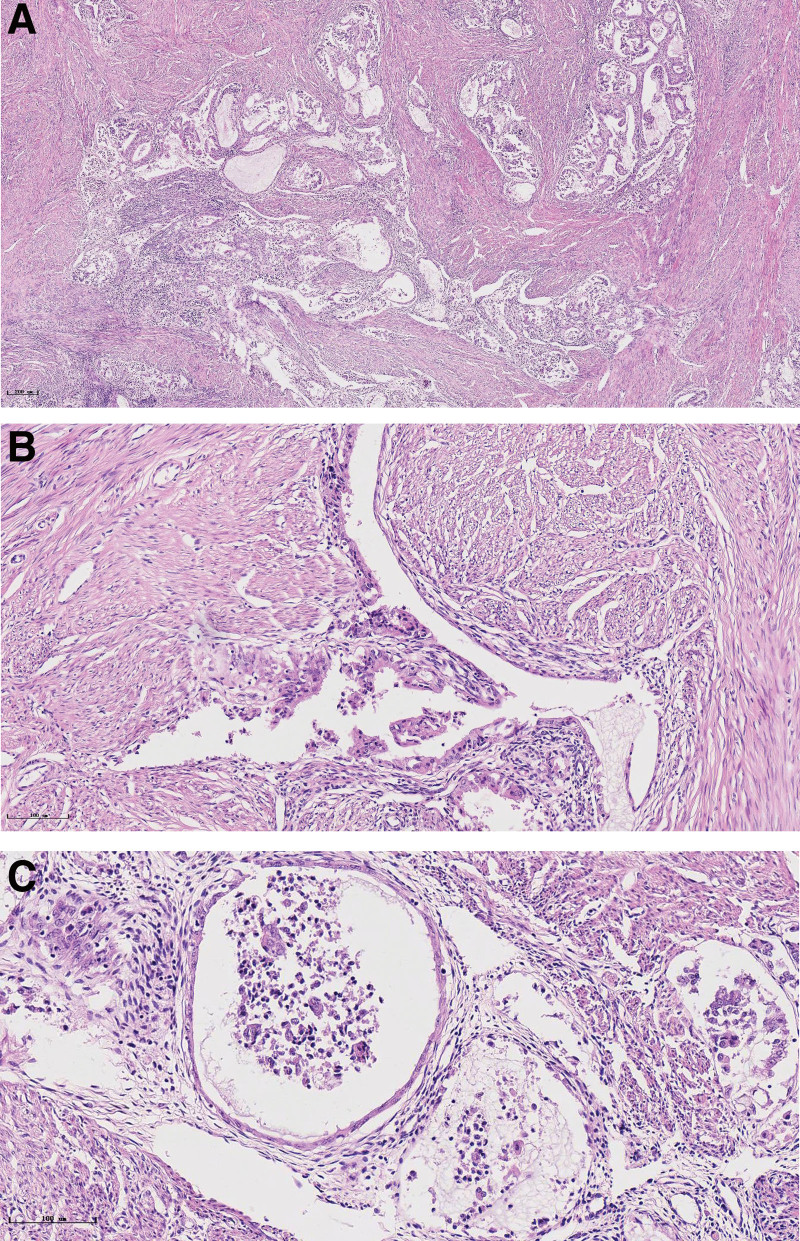
Representative images of MELF pattern in endometrial endometrioid carcinoma (A); elongated gland lined by simple squamous epithelium and columnar epithelium, with lumen containing eosinophilic tumor cells (B); microcystic gland with neutrophilic infiltration (C). HE staining: (A) × 50; (B) × 200; (C) × 400. MELF = microcystic, elongated, and fragmented.

Representative photographs of MELF pattern in endometrial endometrioid carcinoma were presented in Fig. 1. Table 1 presents the clinical characteristics and clinicopathologic parameters with MELF pattern. The frequency of MELF pattern was 17.99% (34/189). The presence of MELF pattern was associated with tumor size (*P* = .003), deep myometrial invasion (*P* < .001), histological grade (*P* < .001), FIGO stage (*P* < .001), LVSI (*P* < .001), and LNM (*P* = .001). There was no significant difference in patient age and cervical stroma involvement between MELF + patients and MELF− patients. The result of tumor marker CA 125 was also related to MELF pattern (*P* < .001). The proportion of adjuvant therapy was significantly higher in MELF pattern (*P* < .001). MELF pattern was present only in low-grade but not in high-grade endometrioid carcinomas.

**Table 1:** Clinical characteristics and clinicopathologic parameters of all study patients.

Parameter	MELF + (n = 34)	MELF− (n = 155)	χ^2^/t	*P*
Age (yrs)	58.03 ± 7.93	52.71 ± 10.50	2.784	.061
Tumor size (cm)	2.12 (2.02)	1.88 (1.86)	−2.980	.003
CA 125 (U/ml)	47.11 (66.60)	15.99 (12.94)	−3.962	<.001
Myometrial invasion				
<1/2	12 (35.29%)	136 (87.74%)	45.152	<.001
≥1/2	22 (64.71%)	19 (12.26%)		
Cervical stroma involvement				
Absent	27 (79.41%)	139 (89.68%)	2.749	.097
Present	7 (20.59%)	16 (10.32%)		
Lymph node metastasis*				
Absent	24 (70.59%)	112 (91.80%)	10.707	.001
Present	10 (29.41%)	10 (8.20%)		
Lymphovascular space invasion				
Absent	10 (29.41%)	125 (80.65%)	35.863	<.001
Present	24 (70.59%)	30 (19.35%)		
Histological grade				
1	5 (14.71%)	69 (44.52%)	20.837	<.001
2	29 (85.29%)	66 (42.58%)		
3	0	20 (12.90%)		
FIGO stage				
StagesI/II	21 (61.76%)	140 (90.32%)	18.019	<.001
StagesIII/IV	13 (38.24%)	15 (9.68%)		
Adjuvant therapy				
No	2 (5.88%)	86 (55.48%)	27.571	<.001
Yes	32 (94.12%)	69 (44.52%)		
Immunophenotype MMR				
Stable	24 (70.59%)	101 (65.16%)	0.367	.545
Instable	10 (29.41%)	54 (34.84%)		
MLH1-PMS2				
Positive	28 (82.35%)	111 (71.61%)	1.653	.199
Negative	6 (17.65%)	44 (28.39%)		
MSH2-MSH6				
Positive	30 (88.24%)	145 (93.55%)	1.148	.284
Negative	4 (11.76%)	10 (6.45%)		
p53				
Wild type	30 (88.24%)	141 (90.97%)	0.242	.623
Mutated	4 (11.76%)	14 (9.03%)		

FIGO= International Federation of Gynecologists and Obstetricians, MELF= microcystic, elongated and fragmented pattern, MMR = mismatch repair.

*Analysis of 156 patients with lymphadenectomy.

Values are expressed as mean ± SD or median (interquartile range) for parametric or non-parametric data respectively.

Table 2 shows the relationship between prognostic factors and lymph node involvement. According to multivariate logistic regression analysis, LVSI [95% confidence interval 1.021–48.485, *P* = .048] was a significant predictor of lymph node involvement. However, MELF pattern was not a significant predictor (95% confidence interval 0.054–2.279, *P* = .400).

**Table 2:** Results of univariate and multivariate analyses of odds ratios in the logistic regression model with lymph node metastasis as the dependent variable.

Variables	Univariate analysis			Multivariate analysis		
	OR	95% CI	*P*	OR	95% CI	*P*
Age (yr)	0.984	0.923–1.119	.579	1.017	0.894–1.183	.738
Tumor size (cm)	0.746	0.804–2.234	.186	1.340	0.448–2.243	.261
MELF	0.351	0.087–2.651	.273	0.480	0.054–2.279	.400
Myometrial invasion	2.916	0.482–17.661	.244	2.904	0.458–18.432	.258
Lymphovascular space invasion	6.785	1.090–42.251	.040	7.035	1.021–48.485	.048
Cervical stroma involvement	2.856	0.395–20.626	.298	4.832	0.176–40.537	.447
Histological grade	1.326	0.060–29.068	.858	0.685	0.722–32.317	.768

CI = confidence interval, MELF = microcystic, elongated and fragmented pattern, OR = odds ratio.

### 3.2. Immunohistochemical findings

**Figure 2.**
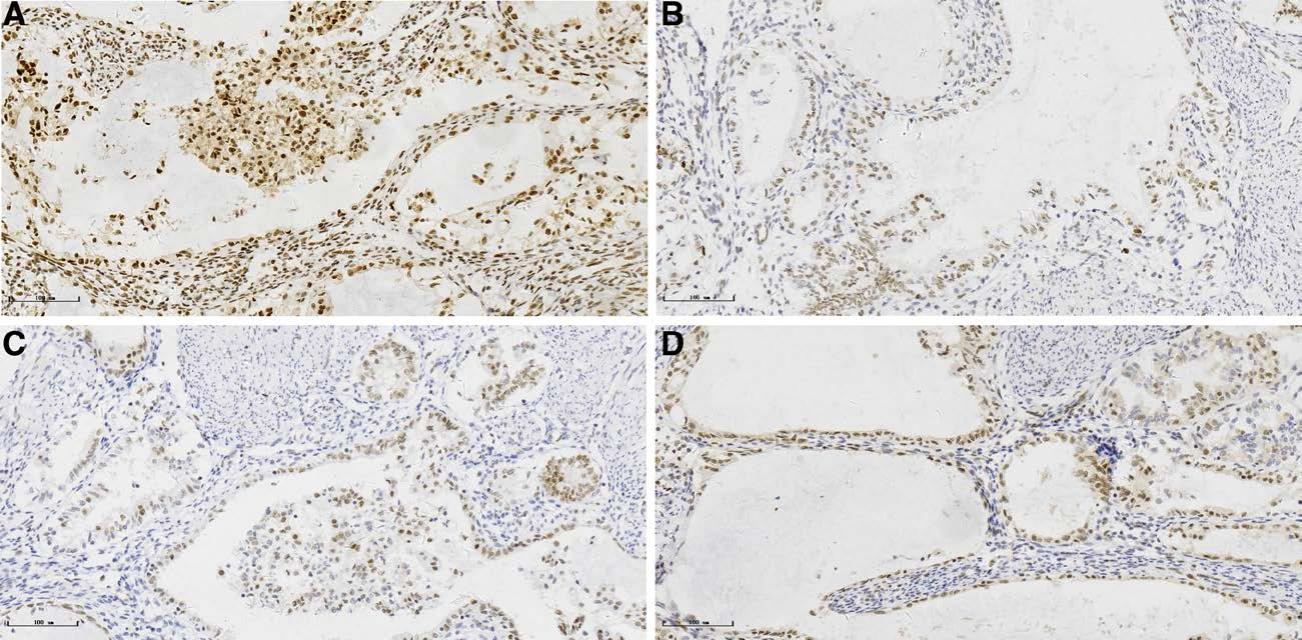
Immunohistochemistry of MELF pattern in endometrial endometrioid carcinoma. MELF-pattern glands are moderate positive for MSH2 (A), strong positive for MSH6 (B), weak positive for MLH1 (C) and weak positive for PMS2 (D). MELF = microcystic, elongated, and fragmented.

Loss of expression for MMR proteins was observed in 10 MELF + cases (29.41%) and 54 MELF− cases (34.84%), respectively. The details regarding type of protein loss were shown in Table 1. However, there was no significant differences between the 2 groups in the distribution of the MMR proteins alterations. Nevertheless, a statistical trend has been observed. Our data showed a higher prevalence of MSH2-MSH6 loss in MELF + group (11.76% in MELF + cases vs 6.45% in MELF− cases) but a higher frequency of MLH1-PMS2 loss in MELF− group (28.39% in MELF− cases vs 17.65 % in MELF + cases) (Fig. 2).

Thirty cases (83.33%) showed a wild-type pattern for p53 in MELF + patients. Among MELF− patients, 141 cases (90.79%) showed a wild-type pattern for p53. There were no significant differences between the 2 groups in the p53 phenotype.

### 3.3. Survival and recurrence

All the 189 patients were followed up for 36.8 ± 8.9 months (18–54 months). Only 1 patient with MELF pattern was diagnosed with vaginal recurrence 28 months after the surgery. She, diagnosed as FIGO stage IIIA, underwent laparoscopic hysterectomy with bilateral salpingo-oophorectomy and pelvic lymphadenectomy, and underwent chemotherapy. She underwent partial upper vaginectomy and radiotherapy for the recurrence. One patient without MELF pattern died of heart failure 6 months after the diagnosis of endometrial cancer. The other patients were followed up with no local recurrence or systemic metastasis occurred.

## 4. Discussion

Endometrial endometrioid carcinomas show various patterns of myometrial invasion. There have been described 5 myoinvasive patterns, respectively diffusely infiltrating, broad front, adenomyosis-like, microcystic, elongated, and fragmented (MELF) glands and adenoma malignum, each having morphological and prognostic particularities. The frequency of MELF pattern was 17.99% in our study. MELF pattern is reported with variable frequencies, ranging between 9.4% and 23.1%.^[3,4,10–12]^ The frequencies fluctuated markedly. It may be that some studies included different pathologic types of endometrial cancer. On the other hand, it may also be due to insufficient understanding of the MELF pattern, which had led to an underestimation. The histologic pattern of myometrial invasion in endometrial endometrioid carcinomas may be a possible predictor for tumor evolution.^[13]^ The biological characters and prognostic significance of MELF pattern remained unclear, although several studies have investigated its clinicopathologic features. Our study showed that MELF pattern was associated with adverse histologic findings such as larger tumor size, deeper myometrial invasion, LVSI and LNM in patients with endometrial endometrioid carcinoma. These findings in our study have been demonstrated in some previous studies. One study showed MELF pattern was associated significantly with larger tumor size, myometrial invasion of more than 50%, advanced FIGO stages, LNM and LVSI, papillary architecture, and mucinous differentiation among the patients with low-grade endometrioid carcinoma.^[12]^ MELF pattern was more common in low-grade endometrioid carcinoma.^[2]^ However, Tresserra F et al observed MELF pattern can be seen in high-grade endometrioid adenocarcinoma of the endometrium.^[14]^ In our study, MELF pattern was found exclusively in low-grade endometrioid carcinomas. Han et al found there was a significant correlation between MELF pattern and cervical stroma involvement.^[15]^ In our study, MELF pattern was not associated with cervical stroma involvement.

The association between MELF pattern and LNM remained uncertain. Several studies proved that MELF pattern was associated significantly with LNM.^[4,10,11,16,17]^ The high probability of LNM in MELF pattern can lead to better therapeutic management, where the role of lymphadenectomy in the surgical management of endometrial cancer remains controversial. Given the favorable evolution in most cases of low-grade endometrial carcinoma, lymphadenectomy is generally avoided because of the serious potential short-term and long-term sequelae, such as lower limb lymphedema, vascular or nerve injury, symptomatic lymphocyst and chylous ascites.^[18]^ In this context, identification of MELF pattern could represent an indication for subsequent lymphadenectomy. Furthermore, sentinel node mapping is increasingly being utilized for endometrial cancer staging.^[19]^ However, there were previous studies demonstrated that MELF was a univariate but not multivariate predictor of LNM.^[5]^ Amy S et al also reported that MELF pattern is not an independent risk factor for LNM or extrauterine metastasis.^[11]^ Our study, in agreement with those studies, showed that patients with MELF pattern were more likely to have LNM than those without MELF pattern (29.41% vs 8.20%), while MELF pattern was statistically not a valuable predictor of LNM due to relatively small sample number.

In our study, only 1 patient with MELF pattern was diagnosed with vaginal recurrence 28 months after the diagnosis of endometrial endometrioid carcinomas. The previous study showed that prognostic factors proven to have an impact on evolution and tumor recurrence were age, histological type, depth of myometrial invasion, histological grade, lymphovascular tumor emboli, tumor size (>2 cm) and metastasis in pelvic lymph nodes.^[20]^ However, no significant differences were reported between the presence of MELF pattern and either disease-free survival or disease-specific survival.^[12,17,21]^ These studies suggested that MELF pattern is a concomitant finding that appears in association with tumor progression. MELF pattern itself may have little impact on prognosis, possibly because of a lack of biological potential causing malignant behavior. However, He D et al reported that in POLE-mutant tumors, MELF pattern invasion was associated with a 15.1-fold increase in tumor recurrence or progression risk whereas this phenomenon was not present in the POLE-wild-type subgroup.^[22]^ Regardless, the implication of MELF pattern in survival and recurrences is unclear. More studies are needed to confirm the prognostic and predictive effect of MELF pattern.

The MMR system is a DNA repair mechanism with the role of maintaining genomic integrity by correcting base substitution mismatches that are generated during DNA replication. MMR deficiency results from either somatic or germline mutations most commonly in the genes MLH1, MSH2, MSH6 and PMS2. MMR deficiency is associated with younger age at diagnosis, endometrioid histotype, a higher proportion with Stage I disease, a higher proportion of LVSI and dedifferentiation.^[23]^ However, Nagle CM et al reported that the risk of endometrial carcinoma is not associated with MMR status.^[24]^ Universal tumor testing for MMR is recommended for all women diagnosed with endometrial cancer to identify those with underlying Lynch syndrome. Among patients with endometrial cancer, the weighted prevalence of Lynch syndrome germline mutations was 15% with deficient IHC staining and 19% with a positive microsatellite instability (MSI) analysis.^[25]^ MMR defects can be easily identifiable with immunohistochemical methods, being generally more easily available and in general inexpensive. Nevertheless, IHC staining quality may show limitations sometimes. According to literature data and considering our laboratory results, we retain that IHC may be considered as a first choice for first-line screening to identify patients for genetic testing and that MSI testing should be performed in situations where IHC is normal and clinical suspicion persists, or IHC is uninterpretable or inconclusive for endometrial endometrioid carcinomas. Besides, our study showed a higher prevalence of MSH2-MSH6 loss in MELF + group (11.76% in MELF + cases vs 6.45% in MELF− cases) but a higher frequency of MLH1-PMS2 loss in MELF− group (28.39% in MELF− cases vs 17.65 % in MELF + cases). Santoro A et al also reported that higher prevalence of MSH2-MSH6 loss in MELF + group and MLH1-PMS2 loss in MELF− group may suggest a different molecular signature.^[26]^ Our study supported the hypothesis of a distinct and specific pattern of MMR altered profile among MSI in MELF− group (MLH1-PMS2) and in MELF + group (MSH2-MSH6).

There could be some limitations of our study: the incidence of MELF pattern might be underestimated because the entire lesion of each tumor was not necessarily sampled in our archival slides. Another limitation may be that the follow-up might be too short to assess the survival and recurrence, and we cannot record the prognosis during longer durations. Additional studies with more patients should be performed to confirm the significance of MELF pattern.

In conclusion, we retrospectively investigated the clinical and clinicopathologic characteristics of MELF pattern in Chinese patients with endometrial endometrioid carcinoma. Although MELF pattern was statistically not a valuable predictor of lymph node metastasis, MELF pattern was associated with adverse histologic findings in endometrial endometrioid carcinomas. The MELF pattern may be important for identifying those patients who need comprehensive staging surgery. Nonetheless, its implication in affecting survival and recurrences is unclear and further larger studies are needed to clarify the exact role of MELF in prognosis and adjuvant therapy.

## Acknowledgements

The authors especially thank Dr Jing Yang for her assistance in analysis of the histopathology.

## Author contributions

**Data curation:** Yuhan Cai.

**Methodology:** Hongyan Guo.

**Writing – original draft:** Jinghua Song.

**Writing – review & editing:** Huajun Li.

## References

[1] Alkushi A Abdul-Rahman ZH Lim P. Description of a novel system for grading of endometrial carcinoma and comparison with existing grading systems. Am J Surg Pathol. 2005;29:295–304. 15725797 10.1097/01.pas.0000152129.81363.d2

[2] Hertel JD Huettner PC Pfeifer JD. Lymphovascular space invasion in microcystic elongated and fragmented (MELF)-pattern well-differentiated endometrioid adenocarcinoma is associated with a higher rate of lymph node metastasis. Int J Gynecol Pathol. 2014;33:127–34. 24487466 10.1097/PGP.0b013e318285657b

[3] Pavlakis K Messini I Vrekoussis T. MELF invasion in endometrial cancer as a risk factor for lymph node metastasis. Histopathol. 2011;58:966–73. 10.1111/j.1365-2559.2011.03802.x 21438907

[4] Dogan Altunpulluk M Kir G Topal CS. The association of the microcystic, elongated, and fragmented (MELF) invasion pattern in endometrial carcinomas with deep myometrial invasion, lymphovascular space invasion and lymph node metastasis. J Obstet Gynaecol. 2015;35:397–402. 25279582 10.3109/01443615.2014.960827

[5] Euscher E Fox P Bassett R. The pattern of myometrial invasion as a predictor of lymph node metastasis or extrauterine disease in low-grade endometrial carcinoma. Am J Surg Pathol. 2013;37:1728–36. 24061515 10.1097/PAS.0b013e318299f2ab PMC3805760

[6] Cree IA White VA Indave BI. Revising the WHO classification: female genital tract tumours. Histopathology. 2020;76:151–6. 31846528 10.1111/his.13977

[7] Quddus MR Sung CJ Zhang C. Minor serous and clear cell components adversely affect prognosis in “mixed-type” endometrial carcinomas: a clinicopathologic study of 36 stage-I cases. Reprod Sci. 2010;17:673–8. 20393071 10.1177/1933719110368433

[8] Lee KR Vacek PM Belinson JL. Traditional and nontraditional histopathologic predictors of recurrence in uterine endometrioid adenocarcinoma. Gynecol Oncol. 1994;54:10–8. 8020830 10.1006/gyno.1994.1158

[9] Murray SK Young RH Scully RE. Unusual epithelial and stromal changes in myoinvasive endometrioid adenocarcinoma: a study of their frequency, associated diagnostic problems, and prognostic significance. Int J Gynecol Pathol. 2003;22:324–33. 14501811 10.1097/01.pgp.0000092161.33490.a9

[10] Park JY Hong D Park JY. Association between morphological patterns of myometrial invasion and cancer stem cell markers in endometrial endometrioid carcinoma. Pathol Oncol Res. 2019;25:123–30. 28990139 10.1007/s12253-017-0320-5

[11] Joehlin-Price AS McHugh KE Stephens JA. The microcystic, elongated, and fragmented (MELF) pattern of invasion: a single institution report of 464 consecutive FIGO grade 1 endometrial endometrioid adenocarcinomas. Am J Surg Pathol. 2017;41:49–55. 27740968 10.1097/PAS.0000000000000754 PMC5159271

[12] Kihara A Yoshida H Watanabe R. Clinicopathologic association and prognostic value of microcystic, elongated, and fragmented (MELF) pattern in endometrial endometrioid carcinoma. Am J Surg Pathol. 2017;41:896–905. 28418994 10.1097/PAS.0000000000000856

[13] Amălinei C Aignătoaei AM Balan RA. Clinicopathological significance and prognostic value of myoinvasive patterns in endometrial endometrioid carcinoma. Rom J Morphol Embryol. 2018;59:13–22. 29940607

[14] Tresserra F Pascual MA Arenas M. MELF pattern in myometrial infiltration in endometrioid adenocarcinoma of the endometrium. A retrospective study of 70 cases. Rev Esp Patol. 2018;51:77–83. 29602378 10.1016/j.patol.2017.10.004

[15] Han H Jiang J. The clinical pathological features and prognostic value of microcystic, elongated and fragmented pattern in endometrioid adenocarcinoma. J Practical Obstet Gynaecol. 2019;35:54–9.

[16] Pavlakis K Rodolakis A Vagios S. Identifiable risk factors for lymph node metastases in grade 1 endometrial carcinoma. Int J Gynecol Cancer. 2017;27:1694–700. 28786874 10.1097/IGC.0000000000001070

[17] Sanci M Güngördük K Gülseren V. MELF pattern for predicting lymph node involvement and survival in Grade I-II endometrioid-type endometrial cancer. Int J Gynecol Pathol. 2018;37:17–21. 28319574 10.1097/PGP.0000000000000370

[18] Ignatov A Ivros S Bozukova M. Systematic lymphadenectomy in early stage endometrial cancer. Arch Gynecol Obstet. 2020;302:231–9. 32430757 10.1007/s00404-020-05600-8

[19] Bogani G Murgia F Ditto A. Sentinel node mapping vs. lymphadenectomy in endometrial cancer: a systematic review and meta-analysis. Gynecol Oncol. 2019;153:676–83. 30952370 10.1016/j.ygyno.2019.03.254

[20] Ballester M Bendifallah S Daraï E. European guidelines (ESMO-ESGO-ESTRO consensus conference) for the management of endometrial cancer. Bull Cancer. 2017;104:1032–8. 29173977 10.1016/j.bulcan.2017.10.006

[21] Prodromidou A Vorgias G Bakogiannis K. MELF pattern of myometrial invasion and role in possible endometrial cancer diagnostic pathway: a systematic review of the literature. Eur J Obstet Gynecol Reprod Biol. 2018;230:147–52. 30286364 10.1016/j.ejogrb.2018.09.036

[22] He D Wang H Dong Y. POLE mutation combined with microcystic, elongated and fragmented (MELF) pattern invasion in endometrial carcinomas might be associated with poor survival in Chinese women. Gynecol Oncol. 2020;159:36–42. 32800323 10.1016/j.ygyno.2020.07.102

[23] Kim SR Pina A Albert A. Does MMR status in endometrial cancer influence response to adjuvant therapy? Gynecol Oncol. 2018;151:76–81. 30172479 10.1016/j.ygyno.2018.08.020

[24] Nagle CM O’Mara TA Tan Y. Endometrial cancer risk and survival by tumor MMR status. J Gynecol Oncol. 2018;29:e39. 29533022 10.3802/jgo.2018.29.e39 PMC5920223

[25] Kahn RM Gordhandas S Maddy BP. Universal endometrial cancer tumor typing: How much has immunohistochemistry, microsatellite instability, and MLH1 methylation improved the diagnosis of Lynch syndrome across the population? Cancer. 2019;125:3172–83. 31150123 10.1002/cncr.32203

[26] Santoro A Angelico G Inzani F. Pathological features, immunoprofile and mismatch repair protein expression status in uterine endometrioid carcinoma: focus on MELF pattern of myoinvasion. Eur J Surg Oncol. 2021;47:338–45. 32788094 10.1016/j.ejso.2020.06.041

